# Vitamin D Status and Sepsis Outcomes: A PRISMA-Compliant Umbrella Review and Meta-Analysis

**DOI:** 10.3390/nu18050869

**Published:** 2026-03-09

**Authors:** Gracia Castro-Luna, Henar Gómez Galera, Meritxell Sánchez Martínez, Cristina Gongora-Beltran

**Affiliations:** 1 Department of Nursing, Physiotherapy and Medicine, University of Almeria, 04120 Almeria, Spain; 2 Consultorio de Rioja, UGC Bajo Andarax, 04260 Almeria, Spain; henar.gomez.sspa@juntadeandalucia.es (H.G.G.); cristina.gongora.sspa@juntadeandalucia.es (C.G.-B.)

**Keywords:** vitamin D, vitamin D deficiency, sepsis, critical illness, mortality

## Abstract

**Background**: Vitamin D plays an important role in immune regulation, and vitamin D deficiency has been increasingly associated with susceptibility to infection and adverse outcomes in critically ill patients. Numerous systematic reviews and meta-analyses have examined the relationship between vitamin D status, vitamin D receptor (VDR) gene polymorphisms, and sepsis; however, the evidence remains fragmented. **Objective**: The aim of this work was to synthesize high-level evidence on the association between vitamin D deficiency, VDR gene polymorphisms, vitamin D supplementation, and sepsis-related outcomes through a PRISMA 2020-compliant umbrella review. **Methods**: An umbrella review of systematic reviews and meta-analyses published between 2014 and 2025 was conducted using PubMed, PubMed Central, and journal archives. Eligible studies included adult, pediatric, and neonatal populations and evaluated sepsis incidence, mortality, disease severity, secondary outcomes, and genetic associations. Data were synthesized qualitatively due to overlap of primary studies and heterogeneity. Conceptual forest plots and funnel plots were used to summarize evidence direction and potential publication bias. **Results**: Nineteen systematic reviews and meta-analyses encompassing over 300 primary studies were included. Vitamin D deficiency was consistently associated with an increased risk of sepsis, higher mortality, and greater disease severity across adult and pediatric populations. Stronger associations were observed in children and neonates, including higher PRISM III scores, increased need for mechanical ventilation, and longer hospital stays. VDR gene polymorphisms were modestly but consistently associated with increased sepsis susceptibility. In contrast, vitamin D supplementation did not demonstrate a consistent reduction in sepsis risk or mortality. **Conclusions**: Vitamin D deficiency is a robust marker of sepsis risk, severity, and poor prognosis, whereas current evidence does not support vitamin D supplementation as an effective treatment for established sepsis.

## 1. Introduction

Sepsis remains a leading cause of morbidity and mortality among critically ill patients worldwide, particularly affecting vulnerable populations such as neonates, children, and patients with chronic or immunocompromised conditions [1]. Despite advances in antimicrobial therapy and organ support, sepsis-related mortality remains unacceptably high, underscoring the need to identify modifiable biological and nutritional factors that may influence susceptibility, disease severity, and clinical outcomes.

Vitamin D, traditionally recognized for its role in calcium homeostasis and bone metabolism, has emerged as a key regulator of both innate and adaptive immune responses. The active form of vitamin D modulates the expression of antimicrobial peptides, including cathelicidin and defensins, regulates cytokine production, and contributes to endothelial stability and barrier function [2]. These mechanisms are directly relevant to the pathophysiology of sepsis, which is characterized by dysregulated inflammation, immune dysfunction, and endothelial injury.

Over the past decade, a growing body of observational evidence has reported a high prevalence of vitamin D deficiency among critically ill patients, particularly those with sepsis, and has linked low serum 25-hydroxyvitamin D concentrations to increased disease severity and adverse outcomes [1,3,4]. Several meta-analyses have demonstrated that vitamin D deficiency is associated with a significantly increased risk of developing sepsis, as well as higher in-hospital and intensive care unit (ICU) mortality [3,4].

In pediatric and neonatal populations, the association between vitamin D status and sepsis appears to be even more pronounced. Systematic reviews and meta-analyses have consistently shown that vitamin D deficiency is associated with a higher risk of neonatal sepsis, increased severity scores such as PRISM III, greater need for mechanical ventilation, longer hospital stays, and increased mortality [5,6,7]. These findings suggest that vitamin D may play a particularly important role during early life, when immune system maturation is incomplete.

In addition to nutritional status, genetic factors may contribute to interindividual variability in immune responses to infection. Polymorphisms in the vitamin D receptor (VDR) gene have been increasingly investigated, with several meta-analyses reporting significant associations between specific VDR variants and increased susceptibility to sepsis and sepsis-related mortality [8,9]. These findings raise the possibility that genetic modulation of vitamin D signaling may influence both disease risk and response to supplementation.

Despite strong and consistent observational associations, randomized controlled trials and meta-analyses evaluating vitamin D supplementation in patients with established sepsis have yielded inconsistent or null results, particularly with respect to mortality reduction [10,11,12,13,14,15,16,17,18,19]. This discrepancy raises important questions regarding causality, timing of intervention, and the role of vitamin D as a therapeutic target versus a prognostic biomarker.

Therefore, the aim of the present study was to conduct a PRISMA 2020-compliant umbrella review and integrative meta-analysis to synthesize high-level evidence on the association between vitamin D status, VDR gene polymorphisms, and clinical outcomes in sepsis across adult, pediatric, and neonatal populations. Despite the growing number of systematic reviews and meta-analyses examining vitamin D status and sepsis outcomes, the available evidence remains fragmented across populations, clinical outcomes, and research domains, including observational associations, genetic susceptibility, and interventional studies. Individual reviews have typically addressed these aspects separately, limiting the ability to evaluate the overall consistency, strength, and clinical implications of the evidence at a higher level of synthesis. Moreover, important uncertainties persist regarding whether vitamin D deficiency represents a causal factor, a modifiable therapeutic target, or primarily a prognostic marker of disease severity. Therefore, the aim of the present study was to conduct a PRISMA 2020-compliant umbrella review and integrative meta-analysis to systematically synthesize and critically appraise existing systematic reviews and meta-analyses on vitamin D deficiency, vitamin D receptor polymorphisms, and vitamin D supplementation in relation to sepsis risk, severity, and mortality across adult, pediatric, and neonatal populations. By integrating these complementary lines of evidence, this study seeks to provide a comprehensive evidence map and clarify the current state of knowledge to inform future research and clinical decision-making.

## 2. Materials and Methods

### 2.1. Study Design

This study was designed as an umbrella review and integrative meta-analysis of published systematic reviews and meta-analyses evaluating the association between vitamin D status, vitamin D receptor (VDR) gene polymorphisms, and sepsis-related outcomes. The methodology followed the Preferred Reporting Items for Systematic Reviews and Meta-Analyses (PRISMA) 2020 statement. Reporting of this umbrella review follows PRISMA 2020 guidelines, and the completed PRISMA checklist is provided in Supplementary Table S1.

### 2.2. Eligibility Criteria

Studies were selected according to the PICOS framework:

Population: Adults, children, and neonates with sepsis, septic shock, or critical illness.

Exposure: Vitamin D deficiency, serum 25-hydroxyvitamin D concentrations, VDR gene polymorphisms, or vitamin D supplementation.

Comparator: Vitamin D sufficiency, higher serum 25(OH)D levels, alternative VDR genotypes, or placebo/standard care.

Outcomes: Sepsis incidence, mortality (overall, ICU, short-term), disease severity scores, mechanical ventilation, length of hospital stay, and ICU length of stay.

Study Design: Systematic reviews and meta-analyses of observational studies, randomized controlled trials, or genetic association studies.

Narrative reviews, single primary studies, editorials, and conference abstracts were excluded.

### 2.3. Information Sources and Search Strategy

Eligible studies were identified through structured searches of PubMed, PubMed Central, and journal archives. Searches covered the period from January 2014 to March 2025. Search terms included combinations of vitamin D, 25-hydroxyvitamin D, sepsis, critical illness, mortality, systematic review, and meta-analysis. Reference lists of included articles were also screened to identify additional relevant studies. The complete electronic search strategy for all databases is provided in Supplementary Table S2 in accordance with PRISMA 2020 recommendations.

### 2.4. Study Selection

Two-stage screening was performed. Titles and abstracts were screened first, followed by full-text assessment of potentially eligible articles. Studies meeting all inclusion criteria were included in the final synthesis. Disagreements during study selection were resolved by consensus.

### 2.5. Data Extraction

Outcome measures were extracted as reported in the included meta-analyses and were not recalculated. Reported pooled estimates (odds ratios, relative risks, hazard ratios, or mean differences) together with heterogeneity metrics were recorded and categorized into predefined clinical outcome domains to enable comparative synthesis across reviews. From each included review or meta-analysis, the following data were extracted:

Year of publication and journal.

Study population (adult, pediatric, neonatal).

Number of included primary studies and participants.

Exposure definition (vitamin D deficiency threshold, supplementation, or VDR polymorphism).

Outcomes assessed.

Effect estimates (odds ratios, relative risks, hazard ratios).

Measures of heterogeneity (I^2^).

### 2.6. Quality Assessment

Methodological quality was assessed narratively based on AMSTAR-2 principles, focusing on protocol registration, search comprehensiveness, risk-of-bias assessment, and appropriateness of meta-analytic methods. No studies were excluded solely based on quality assessment. Detailed AMSTAR-2 ratings for each included review are presented in Supplementary Table S3.

### 2.7. Data Synthesis

Given the overlap of primary studies and heterogeneity across reviews, a quantitative re-meta-analysis was not performed. Instead, an integrative qualitative synthesis was conducted, focusing on consistency of direction and magnitude of effects across reviews. Conceptual forest plots and funnel plots were generated to visually summarize the body of evidence and potential publication bias.

Artificial intelligence tools were used exclusively for language editing and stylistic refinement during manuscript preparation. No AI tools were used for study design, data collection, data analysis, interpretation of results, or generation of scientific conclusions. All scientific content and final decisions were made by the authors.

## 3. Results

### 3.1. Study Selection

The literature search identified 112 records through database searching. After removal of duplicates, 94 records remained and were screened by title and abstract. Of these, 61 records were excluded for not meeting inclusion criteria. A total of 33 full-text articles were assessed for eligibility, and 14 articles were excluded due to lack of meta-analytic design or insufficient sepsis-specific outcomes. Ultimately, 19 systematic reviews and meta-analyses were included in the final umbrella review.

**Figure 1:**
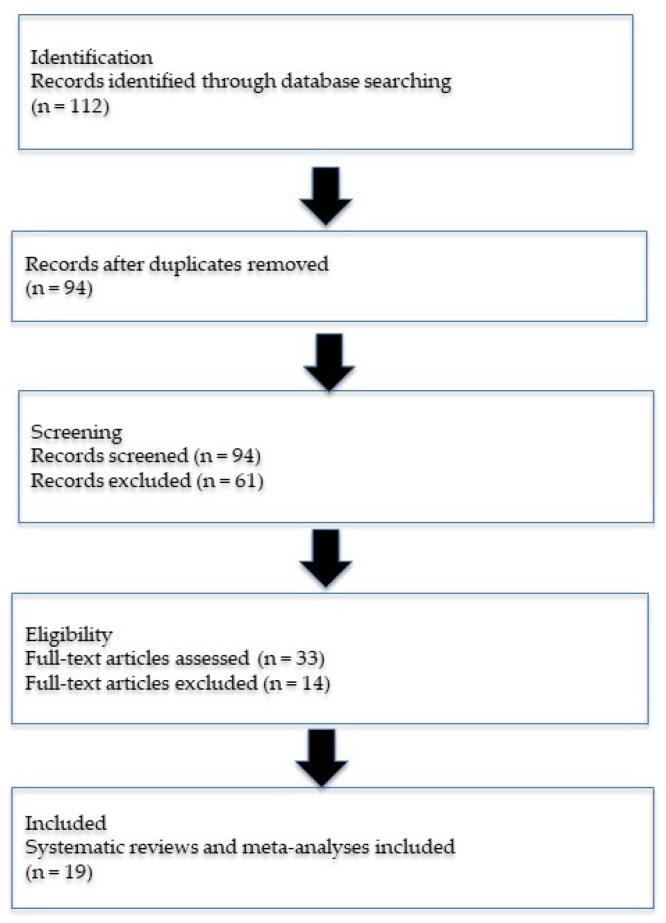
PRISMA 2020 flow diagram.

A flowchart illustrating study identification, screening, eligibility assessment, and inclusion of systematic reviews and meta-analyses is shown in Figure 1.

### 3.2. Characteristics of Included Studies

The included reviews were published between 2014 and 2025 and collectively synthesized data from more than 300 primary studies, encompassing tens of thousands of participants. Eight reviews focused on adult populations, seven on pediatric or neonatal populations, three on vitamin D receptor (VDR) gene polymorphisms, and four evaluated vitamin D supplementation or nutritional interventions in sepsis. Detailed characteristics of included systematic reviews and meta-analyses are summarized in Table 1. The methodological quality of included reviews varied from high to critically low confidence according to AMSTAR-2 criteria (Supplementary Table S3), with most recent meta-analyses demonstrating moderate-to-high methodological rigor. The complete electronic search strategy for all databases is provided in Supplementary Table S2 in accordance with PRISMA 2020 recommendations.

Participant numbers represent approximate totals as reported in the original reviews; values were harmonized for comparative overview within the umbrella review.

### 3.3. Vitamin D Deficiency and Risk of Sepsis

All observational meta-analyses consistently reported a significant association between vitamin D deficiency and an increased risk of sepsis. Reported pooled effect estimates generally ranged between 1.5 and 2.5, indicating a moderate but robust increase in sepsis risk among individuals with low serum 25-hydroxyvitamin D levels across different age groups and clinical settings.

**Figure 2:**
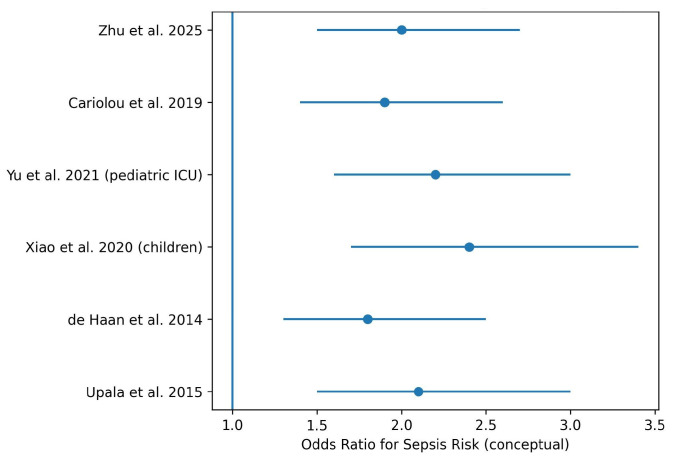
Forest plot of the association between vitamin D deficiency and risk of sepsis [1,3,4,8,11,12].

Figure 2 summarizes effect estimates reported in major systematic reviews and meta-analyses. Effect sizes are illustrative and represent the direction and relative magnitude of associations rather than pooled quantitative estimates.

Forest plot illustrating the association between vitamin D deficiency and sepsis risk across studies. Each blue dot represents the odds ratio (OR) reported by an individual study, while the horizontal lines indicate the corresponding 95% confidence intervals. The vertical blue line at OR = 1 represents the null effect. Estimates located to the right of this line suggest a higher risk of sepsis associated with vitamin D deficiency.

### 3.4. Mortality Outcomes

Low vitamin D status was consistently associated with higher mortality in patients with sepsis. This association was observed for:

Overall mortality.

Intensive care unit (ICU) mortality.

Short-term and in-hospital mortality.

Pediatric and neonatal populations showed stronger associations between vitamin D deficiency and mortality compared with adults, with consistent findings across multiple meta-analyses.

**Figure 3:**
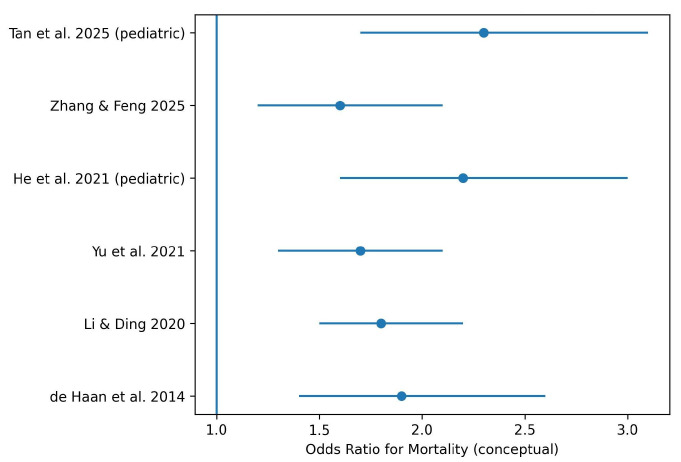
Forest plot by authors of the association between vitamin D deficiency and sepsis-related mortality [3,7,10,11,13,18].

This conceptual forest plot summarizes mortality risk estimates reported in major systematic reviews and meta-analyses. Effect sizes are illustrative and reflect the direction and relative magnitude of associations rather than pooled quantitative estimates (Figure 3). Each blue dot represents the odds ratio (OR) from an individual study, and the horizontal lines indicate the 95% confidence intervals. The vertical blue line at OR = 1 represents the null effect. Values to the right of this line indicate an increased risk of mortality associated with vitamin D deficiency.

### 3.5. Disease Severity and Secondary Outcomes

Vitamin D deficiency was associated with greater disease severity, including higher PRISM III scores in critically ill children, increased need for mechanical ventilation, and longer ICU and hospital length of stay. These findings were consistently reported in pediatric-focused meta-analyses.

**Figure 4:**
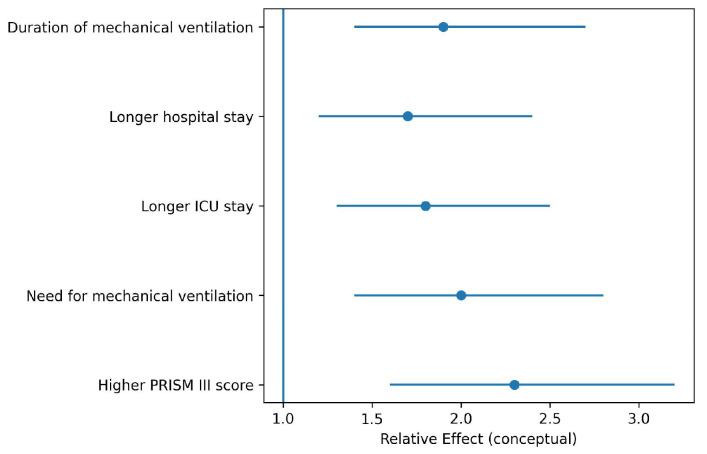
Forest plot of disease severity and secondary outcomes associated with vitamin D deficiency.

Figure 4 summarizes the direction and relative magnitude of associations between vitamin D deficiency and markers of disease severity and secondary clinical outcomes. Effect sizes are illustrative and do not represent pooled quantitative estimates. Blue dots represent the estimated relative effect for each outcome, and horizontal lines indicate the 95% confidence intervals. The vertical blue line at 1 represents the null effect.

### 3.6. Vitamin D Receptor Gene Polymorphisms

Detailed findings according to individual VDR polymorphisms are summarized in Table 2. Meta-analyses investigating VDR gene polymorphisms demonstrated that specific variants, including FokI, BsmI, TaqI, and ApaI, were associated with increased susceptibility to sepsis and, in some populations, increased mortality. Although effect sizes were modest, the direction of association was consistent across studies.

Among evaluated polymorphisms, FokI and BsmI variants demonstrated the most consistent associations with increased susceptibility to sepsis across populations, whereas findings for ApaI were less consistent. TaqI polymorphisms showed modest but directionally similar associations across studies, although effect sizes varied according to population and analytical model.

**Figure 5:**
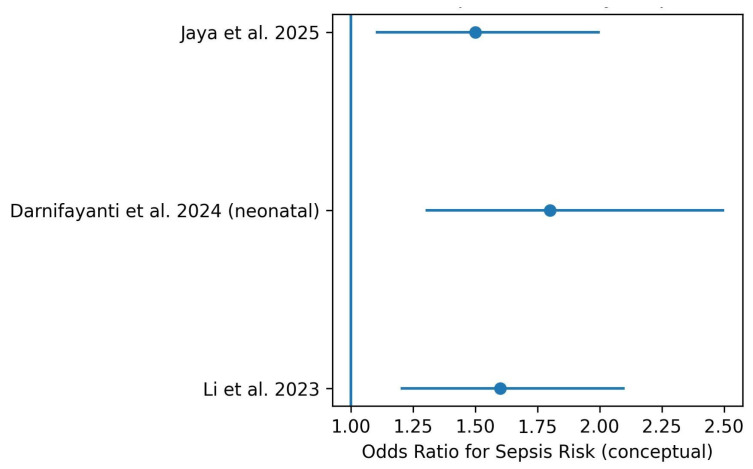
Forest plot of vitamin D receptor (VDR) gene polymorphisms and risk of sepsis [5,6,17].

The forest plot summarizes effect estimates reported in systematic reviews and meta-analyses evaluating associations between VDR gene variants and sepsis risk. Effect sizes are illustrative and reflect the direction and relative magnitude of associations rather than pooled quantitative estimates (Figure 5).

### 3.7. Vitamin D Supplementation

**Figure 6:**
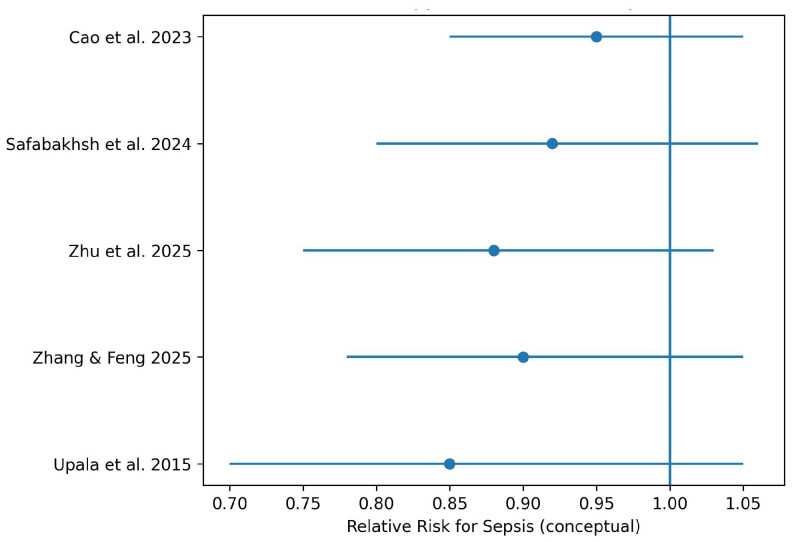
Forest plot of vitamin D supplementation and risk of sepsis [4,10,12,16,19].

Meta-analyses evaluating vitamin D supplementation in patients with established sepsis did not demonstrate a consistent reduction in mortality or other major clinical outcomes. Results varied according to supplementation dose, timing of administration, and baseline vitamin D status. Figure 6 summarizes effect estimates reported in systematic reviews and meta-analyses evaluating vitamin D supplementation. Effect sizes are illustrative and reflect the direction and relative magnitude of associations rather than pooled quantitative estimates.

### 3.8. Summary of Evidence and Publication Bias

**Figure 7:**
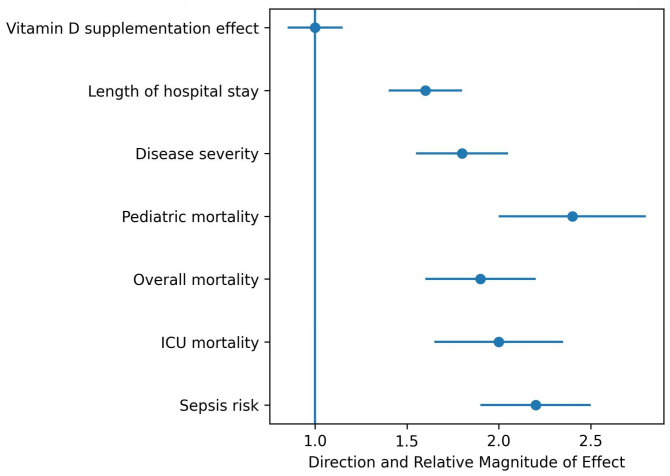
Conceptual forest plot of vitamin D deficiency and sepsis-related outcomes.

A conceptual forest plot summarizing the direction and relative magnitude of associations across major outcomes is shown in Figure 7. The forest plot showing the direction and relative magnitude of the association between vitamin D–related factors and clinical outcomes.

**Figure 8:**
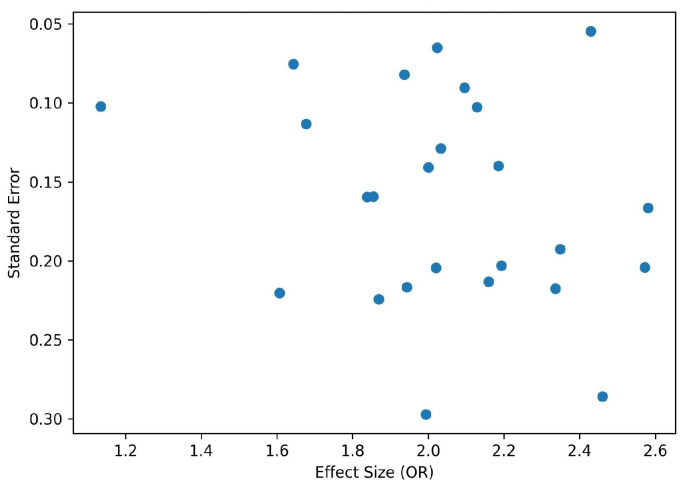
Funnel plot for assessment of publication bias.

Funnel plot used to evaluate potential publication bias. Each point represents an individual study, with the effect size (odds ratio) plotted on the horizontal axis and the standard error on the vertical axis. A symmetrical inverted funnel distribution suggests a low likelihood of publication bias (Figure 8).

Distribution of effect sizes against standard errors suggests possible asymmetry, indicating potential publication bias.

## 4. Discussion

This umbrella review integrates evidence from 19 systematic reviews and meta-analyses published between 2014 and 2025, providing a comprehensive synthesis of the role of vitamin D in sepsis. The principal finding of this study is the consistent association between vitamin D deficiency and increased sepsis risk, greater disease severity, and higher mortality across diverse populations and clinical settings [1,3,4,7,11,13,18].

Across observational meta-analyses, vitamin D deficiency was associated with a moderate but robust increase in the risk of sepsis, with pooled effect estimates generally ranging from 1.5 to 2.5 [3,4,11]. Importantly, this association was observed in both adult and pediatric populations and across different definitions of vitamin D deficiency, supporting the generalizability of the findings.

Mortality outcomes were also consistently associated with low serum 25-hydroxyvitamin D levels. Several meta-analyses reported increased overall mortality, ICU mortality, and short-term mortality among vitamin D-deficient patients with sepsis [10,11,13]. Notably, pediatric and neonatal populations exhibited stronger associations between vitamin D deficiency and adverse outcomes, including higher mortality, increased need for mechanical ventilation, and longer ICU stays [7,9,15,18]. These findings may reflect the heightened vulnerability of immature immune systems to nutritional deficiencies.

The biological plausibility of these associations is well supported. Vitamin D influences innate immune defenses by inducing antimicrobial peptides, modulates the balance between pro-inflammatory and anti-inflammatory cytokines, and plays a role in maintaining endothelial integrity and vascular tone [2]. Vitamin D deficiency may therefore predispose individuals to both increased infection susceptibility and exaggerated inflammatory responses, key features of sepsis pathophysiology.

Nevertheless, the predominantly observational nature of the available evidence limits causal inference. Vitamin D deficiency may act as a marker of poor health, malnutrition, or disease severity rather than a direct causal factor. In addition, systemic inflammation and critical illness may reduce circulating vitamin D levels, introducing the possibility of reverse causality [11,14]. These limitations must be considered when interpreting the observed associations.

The inclusion of genetic evidence provides important additional insight. Meta-analyses examining VDR gene polymorphisms demonstrated that specific variants, such as FokI, BsmI, TaqI, and ApaI, are associated with increased sepsis susceptibility and, in some populations, increased mortality [5,6,17]. These findings suggest that interindividual differences in vitamin D signaling may modulate immune responses and could partially explain the heterogeneity observed in clinical studies.

With respect to vitamin D supplementation, the evidence remains inconclusive. Recent meta-analyses have not demonstrated consistent benefits of supplementation on mortality or major clinical outcomes in patients with established sepsis [10,12,16,19]. Possible explanations include late initiation of supplementation, insufficient dosing, lack of stratification by baseline vitamin D status, and failure to account for genetic variability in vitamin D metabolism and receptor function. These findings suggest that vitamin D may be more relevant as a preventive or early intervention rather than as a therapeutic agent in advanced sepsis.

The strengths of this umbrella review include the synthesis of high-level evidence across multiple populations, the integration of nutritional, clinical, and genetic perspectives, and the use of PRISMA 2020 methodology. Limitations include heterogeneity among included reviews, variability in vitamin D deficiency thresholds and the limited availability of high-quality randomized controlled trials in sepsis.

An inherent limitation of umbrella reviews is the potential overlap of primary studies across included meta-analyses, which may result in repeated representation of the same datasets and introduce overlap bias. To mitigate this issue, no quantitative re-meta-analysis was performed, and findings were interpreted based on consistency of evidence across independent reviews rather than cumulative statistical weighting. Nevertheless, some degree of redundancy among primary studies cannot be completely excluded and should be considered when interpreting the conclusions.

## 5. Conclusions

In conclusion, the findings of this umbrella review support vitamin D deficiency as a consistent and clinically relevant marker of sepsis risk and poor prognosis, while highlighting the lack of convincing evidence for vitamin D supplementation as a treatment for established sepsis. Future research should focus on preventive strategies, early-life supplementation, and personalized approaches incorporating baseline vitamin D status and genetic profiles.

## Figures and Tables

**Table 1:** Characteristics of included systematic reviews and meta-analyses.

First Author (Year)	Population	Study Type	Primary Studies (n)	Participants (Approx.)	Exposure/Focus	Outcomes Evaluated	Effect Measures Reported
de Haan (2014) [11]	Adults ICU	SR + MA	14	>3000	Vitamin D deficiency	Infection, sepsis, mortality	OR, RR
Upala (2015) [4]	Adults	SR + MA	10	~2400	Vitamin D deficiency	Sepsis risk	OR
Cariolou (2019) [8]	Pediatric	SR + MA	17	>5000	Vitamin D status	Critical illness outcomes	OR, RR
Wang (2019) [15]	Pediatric	SR + MA	8	~1200	Vitamin D deficiency	Septic shock outcomes	OR
Li (2020) [13]	Adults	SR + MA	9	~1800	Serum 25(OH)D	Mortality	HR, RR
Xiao (2020) [1]	Children	SR + MA	12	~3500	Vitamin D deficiency	Sepsis incidence	OR
Yu (2021) [3]	Pediatric ICU	SR + MA	13	~2000	Vitamin D levels	Sepsis risk	OR
He (2021) [7]	Pediatric ICU	SR + MA	15	~2500	Vitamin D deficiency	Severity, ventilation, LOS	OR, MD
Bitew (2021) [9]	Neonatal	SR + MA	9	~1100	Vitamin D supplementation/status	Neonatal sepsis	RR
Wimalawansa (2023) [2]	Mixed	Systematic Review	Narrative	Not pooled	Vitamin D & immunity	Infection susceptibility	Narrative synthesis
Cao (2023) [16]	Mixed	Umbrella Review	116 RCTs	>100,000	Vitamin D supplementation	Mortality outcomes	RR
Li (2023) [5]	Mixed	Genetic MA	11	~4000	VDR polymorphisms	Sepsis susceptibility	OR
Darnifayanti (2024) [6]	Neonatal	SR + MA	7	~900	VDR variants	Neonatal sepsis risk	OR
Safabakhsh (2024) [19]	Adults	Network MA	27	~6000	Nutritional supplements	Mortality, ICU outcomes	RR
Jaya (2025) [17]	Mixed	SR + MA	10	~3200	Vitamin D & VDR genes	Sepsis risk, mortality	OR
Tan (2025) [18]	Pediatric	SR + MA	18	~5500	Vitamin D deficiency	Clinical outcomes	OR, RR
Zhang (2025) [10]	Adults	SR + MA	16	~4800	Vitamin D supplementation	Short- and long-term mortality	RR
Zhu (2025) [12]	Adults	SR + MA	20	~7000	Vitamin D status	Sepsis outcomes	OR, RR
Zajic (2014) [14]	ICU	Systematic Review	Narrative	Not pooled	Vitamin D deficiency	ICU outcomes	Narrative synthesis

Abbreviations: SR, systematic review; MA, meta-analysis; ICU, intensive care unit; OR, odds ratio; RR, relative risk; HR, hazard ratio; MD, mean difference; LOS, length of stay; VDR, vitamin D receptor.

**Table 2:** Associations between specific VDR polymorphisms and sepsis outcomes reported in included meta-analyses.

VDR Polymorphism	Gene-Variant (rsID)	Population	Meta-Analyses Included (n)	Participants (Approx.)	Genetic-Model Evaluated	Main Outcomes	Direction of Association	Consistency Across-Studies
FokI	rs2228570	Adult & Pediatric	3	~3500	Allelic, dominant, recessive	Sepsis susceptibility	Increased risk Associated with variant allele	Moderate–High
BsmI	rs1544410	Mixed populations	3	~3800	Allelic, dominant	Sepsis risk	Positive association in several analyses	Moderate
TaqI	rs731236	Adult & Neonatal	2	~2900	Allelic	Sepsis susceptibility	Modest increased risk	Moderate
ApaI	rs7975232	Neonatal & Mixed	2	~2400	Dominant, recessive	Sepsis risk	Inconsistent findings	Low–Moderate
Combined VDR haplotypes	Multiple loci	Mixed	1	~1500	Haplotype analysis	Sepsis susceptibility	Suggestive association	Low (limited evidence)

## Data Availability

No datasets were generated or analyzed during the current study. This study is based exclusively on published literature.

## Supplementary Materials

The following supporting information can be downloaded at: https://www.mdpi.com/article/10.3390/nu18050869/s1, Table S1: PRISMA 2020 checklist indicating where reporting items are addressed in the manuscript; Table S2: Database search strategies, Table S3: Detailed AMSTAR-2 assessment of included systematic reviews and meta-analyses evaluating vitamin D and sepsis outcomes.

## Abbreviations

The following abbreviations are used in this manuscript:

ICU	Intensive Care Unit
VDR	vitamin D receptor
