# Supplementary material for: Vitamin D Status and Sepsis Outcomes: A PRISMA-Compliant Umbrella Review and Meta-Analysis
Source: Nutrients. 2026 Mar 9;18(5):869. doi: 10.3390/nu18050869 (PMC12986698; doi:10.3390/nu18050869)
Supplement: Supplementary file 1 [file nutrients-18-00869-s001.zip › nutrients-4164626-supplementary.pdf]

Table S1. PRISMA 2020 checklist indicating where reporting items are addressed in the manuscript

PRISMA 2020 Checklist

Section & Topic	Item	PRISMA 2020 Checklist Item	Location in Manuscript
**TITLE**	1	Identify the report as a systematic review.	Title page
**ABSTRACT**	2	Structured summary including background, objectives, methods, results, and conclusions.	Abstract
**INTRODUCTION**	3	Describe rationale for the review in context of existing knowledge.	Introduction, paragraphs 1–5
	4	Provide explicit statement of objectives or questions.	End of Introduction
**METHODS**	5	Specify inclusion and exclusion criteria.	Section 2.2 Eligibility Criteria
	6	Specify information sources.	Section 2.3 Information Sources
	7	Present full search strategy.	Section 2.3 + Supplementary Table S2
	8	Describe selection process.	Section 2.4 Study Selection
	9	Describe data collection process.	Section 2.5 Data Extraction
	10	List data items sought.	Section 2.5 Data Extraction
	11	Describe risk of bias assessment methods.	Section 2.6 Quality Assessment
	12	Specify effect measures.	Section 2.5 Data Extraction

Section & Topic	Item	PRISMA 2020 Checklist Item	Location in Manuscript
	13	Describe synthesis methods.	Section 2.7 Data Synthesis
	14	Methods to explore heterogeneity.	Section 2.7 Data Synthesis
	15	Assessment of reporting bias.	Section 2.7 + Figure 8
	16	Certainty assessment methods.	Section 2.6 Quality Assessment (AMSTAR-2)
**RESULTS**	17	Study selection results with flow diagram.	Section 3.1 + Figure 1 (PRISMA Flowchart)
	18	Characteristics of included studies.	Section 3.2
	19	Risk of bias results.	Section 3.2 + Supplementary Table S1
	20	Results of individual studies.	Sections 3.3–3.7
	21	Results of syntheses.	Sections 3.3–3.8
	22	Reporting bias assessment results.	Section 3.8 + Figure 8
	23	Certainty of evidence.	Section 3.2 + Discussion
**DISCUSSION**	24a	General interpretation of results.	Discussion, paragraphs 1–3
	24b	Limitations of evidence.	Discussion, limitations paragraph
	24c	Limitations of review processes.	Discussion, limitations paragraph
	24d	Implications for practice and research.	Discussion final paragraphs
**OTHER INFORMATION**	25	Registration and protocol.	Methods (Study Design section)

Section & Topic	Item	PRISMA 2020 Checklist Item	Location in Manuscript
	26	Support/funding sources.	Funding Statement
	27	Competing interests declared.	Conflicts of Interest
	28	Availability of data/materials.	Data Availability Statement

---

This checklist was completed according to the **PRISMA 2020 Statement**. Page numbers and sections refer to the revised manuscript submitted for peer review.

Table S2. Database search strategies

Database: PubMed (MEDLINE)

Component	Search Terms
Concept 1 – Vitamin D	("vitamin D"[Title/Abstract] OR "25-hydroxyvitamin D"[Title/Abstract] OR "25(OH)D"[Title/Abstract] OR "cholecalciferol"[Title/Abstract] OR "ergocalciferol"[Title/Abstract] OR "vitamin D deficiency"[Title/Abstract])
Concept 2 – Sepsis / Critical illness	("sepsis"[Title/Abstract] OR "septic shock"[Title/Abstract] OR "critical illness"[Title/Abstract] OR "intensive care"[Title/Abstract] OR "ICU"[Title/Abstract])
Concept 3 – Evidence synthesis	("systematic review"[Publication Type] OR "systematic review"[Title/Abstract] OR "meta-analysis"[Publication Type] OR "meta-analysis"[Title/Abstract])
Boolean combination	(#1 AND #2 AND #3)
Filters applied	Humans; English language; Publication date: January 1, 2014 – March 31, 2025
Final search string	(("vitamin D"[Title/Abstract] OR "25-hydroxyvitamin D"[Title/Abstract] OR "25(OH)D"[Title/Abstract] OR "cholecalciferol"[Title/Abstract] OR "ergocalciferol"[Title/Abstract] OR "vitamin D deficiency"[Title/Abstract]) AND ("sepsis"[Title/Abstract] OR "septic shock"[Title/Abstract] OR "critical illness"[Title/Abstract] OR "intensive care"[Title/Abstract] OR "ICU"[Title/Abstract]) AND ("systematic review"[Publication Type] OR "meta-analysis"[Publication Type] OR "systematic review"[Title/Abstract] OR "meta-analysis"[Title/Abstract]))
Date of last search	March 31, 2025
Records retrieved	112

Database: PubMed Central (PMC)

Component	Search Terms
Search query	("vitamin D" AND sepsis AND ("systematic review" OR "meta-analysis"))
Fields searched	Full text and metadata
Filters applied	English language; 2014–2025
Screening method	Title/abstract screening followed by full-text eligibility assessment
Date of last search	March 31, 2025
Records retrieved	Included within combined screening results

Additional Search Procedures

Procedure	Description
Reference list screening	Reference lists of all included systematic reviews and meta-analyses were manually screened to identify additional eligible studies.
Duplicate removal	Duplicates were removed prior to screening using manual verification.
Screening process	Two-stage screening (title/abstract followed by full text) performed according to predefined eligibility criteria.
Reviewers	Study selection conducted by two independent reviewers with consensus resolution.

The search strategy was developed to comply with PRISMA 2020 reporting recommendations, ensuring reproducibility and transparency. Database-specific adaptations were applied where necessary while preserving conceptual equivalence across searches.

Table S3: Detailed AMSTAR-2 assessment of included systematic reviews and meta-analyses evaluating vitamin D and sepsis outcomes

**Study (First Author, Year)**	**PICO Defined (1)**	**Protocol Registered (2)***	**Study Design Justified (3)**	**Comprehensive Search (4)***	**Study Selection in Duplicate (5)**	**Data Extraction in Duplicate (6)**	**List of Excluded Studies (7)***	**Study Characteristics (8)**	**Risk of Bias Assessed (9)***	**Funding Sources Reported (10)**	**Appropriate Meta-analysis (11)***	**RoB Considered in Interpretation (13)***	**Publication Bias (15)***	**COI Reported (16)**	**Overall Confidence**
de Haan 2014	Y	PY	Y	Y	Y	Y	N	Y	Y	Y	Y	Y	Y	Y	**High**
Upala 2015	Y	N	Y	Y	Y	Y	N	Y	Y	Y	Y	PY	Y	Y	**Moderate**
Cariolou 2019	Y	Y	Y	Y	Y	Y	Y	Y	Y	Y	Y	Y	Y	Y	**High**
Wang 2019	Y	N	Y	Y	PY	PY	N	Y	PY	Y	Y	PY	Y	Y	**Moderate**
Li 2020	Y	N	Y	Y	Y	Y	N	Y	Y	Y	Y	PY	Y	Y	**Moderate**
Xiao 2020	Y	Y	Y	Y	Y	Y	Y	Y	Y	Y	Y	Y	Y	Y	**High**
Yu 2021	Y	Y	Y	Y	Y	Y	Y	Y	Y	Y	Y	Y	Y	Y	**High**
He 2021	Y	N	Y	Y	PY	PY	N	Y	PY	N	Y	PY	N	Y	**Low**
Bitew 2021	Y	Y	Y	Y	Y	Y	Y	Y	Y	Y	Y	Y	Y	Y	**High**
Wimalawansa 2023	Y	N	N	PY	N	N	N	PY	N	N	NA	N	N	Y	**Critically Low**
Cao 2023	Y	Y	Y	Y	Y	Y	Y	Y	Y	Y	Y	Y	Y	Y	**High**
Li Q 2023	Y	Y	Y	Y	Y	Y	Y	Y	Y	Y	Y	Y	Y	Y	**High**
Darnifayanti 2024	Y	Y	Y	Y	Y	Y	Y	Y	Y	Y	Y	Y	Y	Y	**High**
Safabakhsh 2024	Y	Y	Y	Y	Y	Y	Y	Y	Y	Y	Y	Y	Y	Y	**High**

Table S3: Detailed AMSTAR-2 assessment of included systematic reviews and meta-analyses evaluating vitamin D and sepsis outcomes

Jaya 2025	Y	N	Y	Y	Y	Y	N	Y	Y	Y	Y	PY	Y	Y	**Moderate**
Tan 2025	Y	Y	Y	Y	Y	Y	Y	Y	Y	Y	Y	Y	Y	Y	**High**
Zhang 2025	Y	Y	Y	Y	Y	Y	Y	Y	Y	Y	Y	Y	Y	Y	**High**
Zhu 2025	Y	Y	Y	Y	Y	Y	Y	Y	Y	Y	Y	Y	Y	Y	**High**
Zajic 2014	Y	N	N	PY	N	N	N	PY	N	N	NA	N	N	Y	**Critically Low**

*Critical AMSTAR-2 domains. **Y** = Yes.**PY** = Partial Yes.**N** = No.**NA** = Not applicable.**Critical domains (AMSTAR-2):**Items **2, 4, 7, 9, 11, 13, 15.** Overall confidence ratings were assigned according to AMSTAR-2 guidance (Shea et al., 2017):**High:** No or one non-critical Weakness .**Moderate:** >1 non-critical weakness.**Low:** One critical flaw.**Critically Low:** >1 critical flaw

Critical domains included protocol registration, literature search adequacy, justification of excluded studies, risk-of-bias assessment, appropriateness of meta-analysis, consideration of bias in interpretation, and publication bias assessment.
